# In-hospital and 30-day major adverse cardiac events in patients referred for ST-segment elevation myocardial infarction in Dhaka, Bangladesh

**DOI:** 10.1186/s12872-021-01896-9

**Published:** 2021-02-10

**Authors:** Zubair Akhtar, Mohammad Abdul Aleem, Probir Kumar Ghosh, A. K. M. Monwarul Islam, Fahmida Chowdhury, C. Raina MacIntyre, Ole Fröbert

**Affiliations:** 1 Programme for Emerging Infections, International Center for Diarrhoeal Diseases, Bangladesh (icddr,b), 68 Shaheed Tajuddin Ahmed Sarani, Mohakhali, Dhaka, 1212 Bangladesh; 2 Faculty of Medicine, University of New South Wales, Sydney, Australia; 3 Department of Cardiology, National Institute of Cardiovascular Diseases Dhaka (NICVD), Dhaka, Bangladesh; 4 Department of Cardiology, Faculty of Health, Örebro University, Örebro, Sweden

**Keywords:** STEMI, MACE, Low-income setting, Urban setting

## Abstract

**Background:**

There is a paucity of data regarding acute phase (in-hospital and 30-day) major adverse cardiac events (MACE) following ST-segment elevation myocardial infarction (STEMI) in Bangladesh. This study aimed to document MACE during the acute phase post-STEMI to provide information.

**Methods:**

We enrolled STEMI patients of the National Institute of Cardiovascular Disease, Dhaka, Bangladesh, from August 2017 to October 2018 and followed up through 30 days post-discharge for MACE, defined as the composite of all-cause death, myocardial infarction, and coronary revascularization. Demographic information, cardiovascular risk factors, and clinical data were registered in a case report form. The Cox proportional hazard model was used for univariate and multivariate analysis to identify potential risk factors for MACE.

**Results:**

A total of 601 patients, mean age 51.6 ± 10.3 years, 93% male, were enrolled. The mean duration of hospital stay was 3.8 ± 2.4 days. We found 37 patients (6.2%) to experience an in-hospital event, and 45 (7.5%) events occurred within the 30 days post-discharge. In univariate analysis, a significantly increased risk of developing 30-day MACE was observed in patients with more than 12 years of formal education, diabetes mellitus, or a previous diagnosis of heart failure. In a multivariate analysis, the risk of developing 30-day MACE was increased in patients with heart failure (hazard ratio = 4.65; 95% CI 1.64–13.23).

**Conclusions:**

A high risk of in-hospital and 30-day MACE in patients with STEMI exists in Bangladesh. Additional resources should be allocated providing guideline-recommended treatment for patients with myocardial infarction in Bangladesh.

## Background

Acute coronary syndrome is among the leading causes of morbidity and mortality globally [1], but, while the incidence and mortality rates are declining in most developed nations, they are on the rise in developing countries [2, 3]. The decline may be attributed to increased adherence to treatment guidelines and changes in lifestyle and behavior [4–7]. ST-segment elevation myocardial infarction (STEMI) is the most common acute manifestation of coronary artery disease [8], accounting for approximately one-third of acute coronary syndromes in both developed and developing countries [9, 10]. In economically disadvantaged areas like Bangladesh, the prevalence of coronary artery disease and STEMI remains mostly unknown, and only small scale epidemiological surveys provide evidence of its increase [11].

Myocardial infarction is two to three times more common in patients who have survived an earlier STEMI than in the general population [12]. Older age, no revascularization procedure, and comorbidities have been identified as significant risk factors for recurrence [12]. Most studies of post-STEMI outcomes focus on the acute phase, in-hospital and 30 days post-discharge, after the index event [13, 14]. A literature search produced only a single study from Bangladesh describing post-STEMI outcomes as a composite of major adverse cardiac events (MACE) in a rural setting [15].

In a resource-limited setting, it is not always feasible to adopt the best treatment strategies for the management of STEMI. Differences in epidemiological, as well as clinical factors, may contribute to greater risk of adverse events following STEMI [16, 17] that can potentially impact patient-specific outcomes [18]. This situation exists even in the large tertiary care cardiac hospital in the capital city of Dhaka, as STEMI patients referred from throughout Bangladesh receive treatment here. Bangladesh, with rapidly progressing urbanization, is undergoing a demographic and epidemiological transition from infectious diseases to non-communicable diseases as primary areas of concern [19, 20]. Common behavioural, metabolic, and physiological risk factors of coronary artery disease are prevalent in the Bangladesh population [21, 22]. In light of this transition, and considering the paucity of in-hospital and 30-day MACE data in urban locations in Bangladesh, we aimed to determine MACE rates in the acute phase post-STEMI for evidence-based guidance to inform the mobilization of resources for therapeutic strategies in STEMI.

## Methods

We conducted a prospective longitudinal observational study at the National Institute of Cardiovascular Diseases (NICVD), Dhaka, from August 2017 through October 2018. The NICVD is the largest public tertiary care cardiac hospital in Bangladesh, managing patients with cardiovascular disorders from throughout the country. Study-appointed physicians reviewed NICVD hospital admission records and visited patients admitted to the cardiology wards to identify potential subjects aged ≥ 18 years hospitalized with a STEMI. STEMI diagnosis was confirmed based on ST-segment elevation in the electrocardiogram in the hospital records. Informed written consent to participate in the study was obtained for collection of extensive baseline and outcome information. The study was approved by the icddr,b institutional review board prior to enrolling participants.

### Data collection

Enrolled patients were followed up during hospitalization and for 30 days post-discharge. Study physicians recorded sociodemographic data, cardiovascular risk factors, and clinical data on a case report form. They also extracted data related to medical history from the medical records of the patient records file and verified through clinical examination. A respondent was considered hypertensive when the average of two or more diastolic blood pressure (BP) measurements on at least two subsequent visits was ≥ 90 mm Hg or when the average of multiple systolic BP readings on two or more subsequent visits was consistently ≥ 140 mm Hg in the hospital records [23]. A patient was confirmed to be diabetic based on either of the laboratory findings of plasma glucose: HbA1c ≥ 48 mmol/mol; random plasma glucose ≥ 11.1 mmol/l; fasting plasma glucose ≥ 7.0 mmol/l or oral glucose tolerance test 2-h glucose in venous plasma glucose ≥ 11.1 mmol/l [24]. A respondent was diagnosed with dyslipidemia according to American heart association’s classification corresponding to the a total cholesterol > 5.2 mmol/l (200 mg/dl) or low-density lipoprotein (LDL) > 3.4 mmol/l (130 mg/dl), high-density lipoprotein (HDL) < 0.9 mmol/l (35 mg/dl), or triglycerides > 1.7 mmol/l (150 mg/dl) or a combination thereof [25]. Body mass index (BMI) was calculated by body weight measured in kilograms divided by height in meters squared [26]. On day 31, following discharge from hospital, study physicians made calls to subjects/family members to record any MACE during the past 30 days. A MACE was defined as all-cause death, non-fatal myocardial infarction, or a revascularization procedure including Percutaneous coronary intervention (PCI) or Cronary artery bypass grafting (CABG) [27]. Similar to a previously published study, if more than one MACE occurred during the follow-up period, the most severe endpoint (all-cause death > myocardial infarction > revascularization) was selected for the 30-day MACE analysis [28]. Unplanned revascularizations were only considered for MACE.

### Data analysis

Sociodemographic information and cardiovascular risk factors such as underlying chronic conditions, smoking, family history of cardiovascular disease, previous coronary revascularization procedures, and heart failure were summarized using descriptive statistics. Data of access to water, sanitation, hygiene, and characteristics of housing were collected to classify the wealth index [29] using a principal component analysis [30]. Clinical data including symptoms, cardiac troponin I (cTn-I) level at admission, and location of STEMI based on electrocardiogram/echocardiogram findings were summarized using descriptive statistics.

Pearson’s χ^2^ tests were used to analyse categorical variables, and non-parametric Wilcoxon rank-sum tests were conducted for continuous variables in patients with or without events in the 30-day follow-up period. The event rates and 95% confidence intervals (CI) were tabulated for the in-hospital and post-discharge 30-day follow-up periods.

Univariate and multivariate Cox regression models were used to estimate risk factors for events. Hazard ratios (HR) and the corresponding 95% CI adjusted for covariates were calculated. Based on literature review and clinical input, 12 risk factors were included in analysis: age, sex, wealth index, education level, location of residence (urban/rural), hypertension, diabetes mellitus (DM), dyslipidemia, previous myocardial infarction, tobacco use, family history of cardiovascular disease, and obesity. Covariates that were significant in the univariate analyses at the *p* ≤ 0.20 level were included in the multivariable model. A goodness-of-fit test of the multivariable model was conducted, and the *p* values from Wald tests of the individual variables were used to identify variables that could be excluded from the model to remove any residual effect. Based on the goodness-of-fit test, seven variables were included in the final Cox regression model. The HR for univariate and multivariate models, together with the respective 95% CI, are reported. Among the seven variables in the multivariate analysis, corresponding adjusted HRs were not reported for confounding variables of age and education. A *p* value ≤ 0.05 was considered significant. All analyses were performed using Stata v. 13 (StataCorp LP, College Station, TX, USA).

## Results

From August 2017 through October 2018, 601 patients, mean age 51.6 [SD $\pm$ 10.3] years, range 24–80, 93% (559) male, were diagnosed with STEMI based on clinical presentation at admission and electrocardiogram (ECG) findings and included in the study. No patient was lost to follow-up. Baseline data are presented in Table 1. Two-thirds (389/601) of patients had a family history of cardiovascular disease and 25% (148/601) had diabetes mellitus. Post-MI discharge, 95% (573/601) of patients were prescribed antiplatelet agents like acetylsalicylic acid (ASA) and P2Y12 inhibitors, 90% (540/601) were prescribed statins, 62% (371/601) were prescribed nitrates, 51% (305/601) were prescribed angiotensin-converting enzyme (ACE) inhibitors and 44% (266/601) were prescribed β-blockers (Table 1).

**Table 1:** Baseline characteristics of 601 STEMI patients in Dhaka, Bangladesh (August 2017–October 2018)

Characteristics	Number (%)
Age (years)	
Mean age (SD)	51.6 (± 10.3)
< 40	75 (12.5)
40–64	450 (74.9)
≥ 65	76 (12.7)
Sex	
Male	559 (93.0)
Location of residence	
Rural	275 (45.8)
Urban	326 (54.2)
Education, years of school attendance	
None	154 (25.6)
1–5	249 (41.4)
6–10	60 (10.0)
11–12	57 (9.5)
≥ 13	81 (13.5)
Wealth Index [29]	
Poorest	139 (23.1)
Poorer	107 (17.8)
Middle	122 (20.3)
Wealthier	163 (27.1)
Wealthiest	70 (11.7)
Medical history	
Hypertension	230 (38.3)
Diabetes mellitus	148 (24.6)
Dyslipidemia	60 (10.0)
Family history of CVD	389 (64.7)
Body Mass Index > 25	212 (37.3)
Currently smoking	410 (68.2)
Smokeless tobacco use	56 (12.2)
Alcohol consumption	12 (2.0)
Previous MI	54 (9.0)
Previous heart failure	11 (1.8)
Previous revascularization	8 (1.3)
Discharge medications	
ASA + P2Y12 inhibitor combination	573 (95.3)
Statins	540 (89.9)
Nitrates	371 (61.7)
ACE inhibitors	305 (50.7)
β-blockers	266 (44.3)
Anti-diabetic agents	75 (12.5)

*ACE inhibitor* Angiotensin converting enzyme inhibitor, *ASA* acetylsalicylic acid, *CVD* cardiovascular disease, *MI* myocardial infarction, *SD* standard deviation

Presenting symptoms at hospital are listed in Table 2. An ECG was conducted in all cases. Cardiac troponin I (cTn-I) was not available for 67% (404/601) of cases; however, among those in which it was assessed, 84% (165/197) showed values above the 99th percentile of the upper reference limit. Significantly higher mean values of cTn-I were observed in patients who developed MACE during the 30-day follow-up period compared to patients who did not experience MACE (37.3 ng/ml [± SD 43.4] vs. 19.4 ng/ml [± SD 33.7], *p* = 0.002). Based on ECG/echocardiogram, the most common location of infarct was in the inferior (47%, 283/601) followed by the anterior (27%, 165/601) heart wall.

**Table 2:** Characteristics of STEMI patients at presentation in Dhaka, Bangladesh (August 2017–October 2018)

Characteristics	MACE n = 80	Without MACE n = 521	*p* value
Symptoms n (%)			
Chest discomfort	78 (97.5)	505 (96.9)	0.780
Dyspnea	41 (51.3)	181 (34.7)	**0.004**
Sweating	63 (78.8)	413 (79.3)	0.915
Nausea	43 (53.8)	245 (47.0)	0.262
Vomiting	31 (38.8)	197 (37.8)	0.872
Fainting	6 (7.5)	29 (5.6)	0.492
Troponin I level at admission^a^			
Mean Troponin I, ng/ml (SD)	37.3 (43.4)	19.4 (33.7)	**0.002**
Not elevated, n (%)	2 (2.5)	30 (5.8)	Ref
Elevated, n (%)	27 (33.8)	138 (26.6)	0.157
Not available, n (%)	51 (63.8)	353 (67.8)	–
Location of STEMI n (%)			
Anterior	25 (31.3)	140 (26.9)	Ref
Anteroseptal	10 (12.5)	79 (15.2)	0.383
Inferior	36 (45.0)	247 (47.4)	0.467
Lateral	2 (2.5)	9 (1.7)	0.775
Other	7 (8.8)	46 (8.8)	0.718

Theh bold font was used to indicate that the value is statistically significant, i.e. *p* < 0.005

^a^n = 197 subjects had Troponin I level assessed

The mean duration of hospital stay was 3.8 (± SD, 2.4) days. Hospitalization of patients who experienced in-hospital MACE was significantly longer than recorded for those who experienced post-discharge 30-day MACE (5.6 days, [± SD 4.5] vs. 3.7 days, [± SD 2.1]; *p* = 0.022). Thirty-seven (6.2%; 95% CI 4.2–8.1) in-hospital events included 19 (3.2%) all-cause deaths and 18 (3.0%) unplanned revascularization procedures. Within 30 days post-discharge, 45 (7.5%; 95% CI 5.4–9.6) MACE were recorded, including revascularization procedures (PCI or CABG) in 26 (4.3%) patients, 15 all-cause deaths (2.5%), and 4 (0.7%) recurrent MIs. Two patients undergoing unplanned revascularization during the hospital stay died during the 30-day follow-up period. In total, 80 MACE occurred during the in-hospital and 30-day follow-up, i.e. 13.3% (80/601) of all patients experienced a MACE (Table 3).

**Table 3:** In-hospital and 30-day MACE in STEMI patients in Dhaka, Bangladesh (August 2017–October 2018)

MACE	In hospital n (%)	30 day n (%)	Total
Overall	37 (6.2) (95% CI 4.2–8.1)	45 (7.5) (95% CI 5.4–9.6)	80^a^ (13.3) (95% CI 10.6–16.0)
All-cause deaths	19 (3.2)	15 (2.5)	34 (5.7)
Revascularization	18 (3.0)	26 (4.3)	44 (7.3)
Recurrent MI	0	4 (0.7)	4 (0.7)

^a^Two in-hopital MACE cases experienced more severe MACE post-discharge

**Fig. 1:**
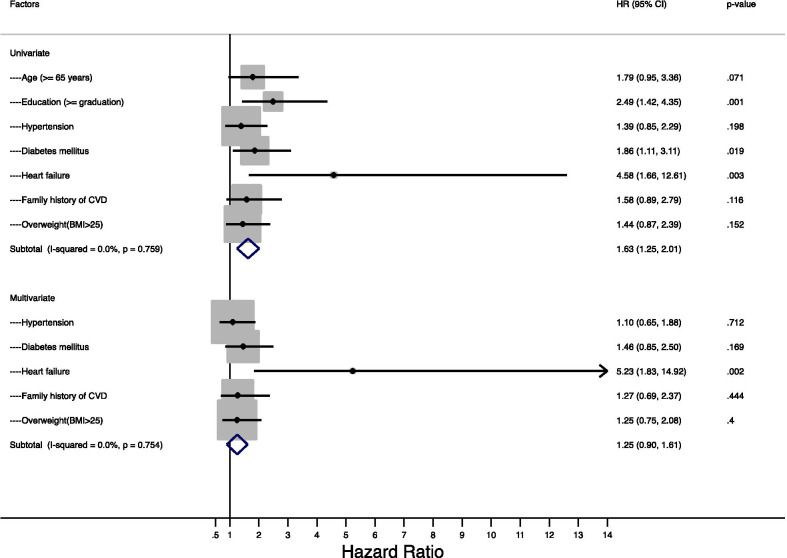
Forest Plot: association between factors and 30-day MACE within subgroups of STEMI patients in Dhaka, Bangladesh (2017–2018)

The univariate analysis revealed significantly increased risk of MACE in patients with greater than 12 years of education, diabetes mellitus, or a previous history of heart failure. We also observed a numerically elevated risk (HR = 1.79; 95% CI 0.95–3.36) in patients ≥ 65 years, but this did not reach statistical significance. In multivariate analysis, after adjusting for age and education, heart failure (HR = 5.23; 95% CI 1.83–14.92) remained a significant risk factor for 30-day MACE (Fig. 1).

## Discussion

**Fig. 2:**
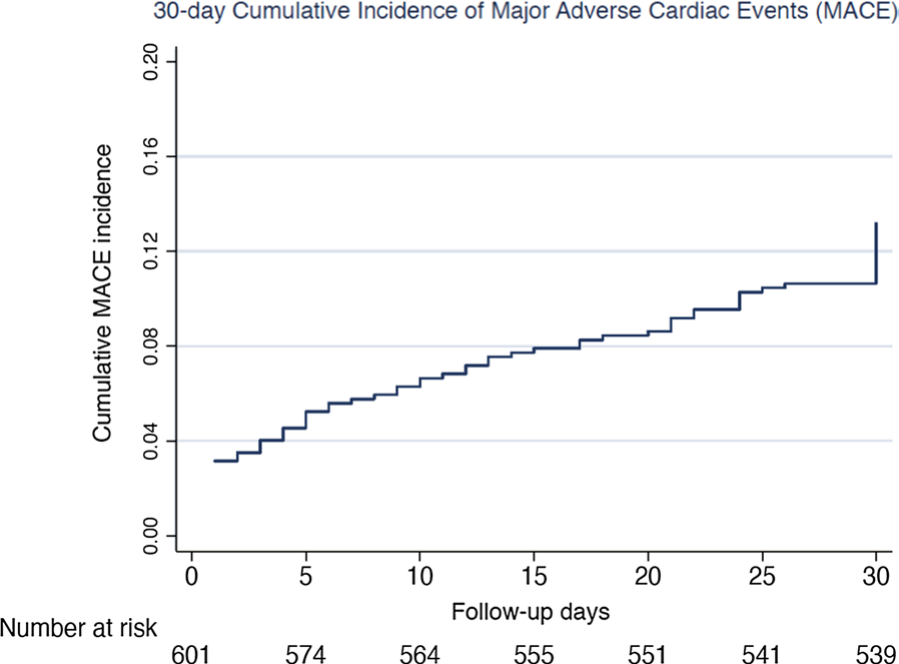
Cumulative incidence of in-hospital and 30-day MACE in STEMI patients in Dhaka, Bangladesh (August 2017–October 2018)

Our prospective study from the largest tertiary cardiac hospital in Bangladesh revealed that 13% of patients admitted with STEMI experienced a MACE within the 30 days post-discharge (Fig. 2). This finding is lower than the 23% MACE within 30 days post-STEMI found in a study in rural Bangladesh [15].

Our 30-day MACE rate following STEMI is much higher compared to reports from the Netherlands (3%), India (5%), and Brazil (10%) [31–33]. The in-hospital MACE rate was lower in our study population compared with results of recent studies from China (12%), Canada (9%), and India (8%) [32, 34, 35]. We were, however, unable to compare our results with regional data because of the paucity of data available of in-hospital MACE in Bangladesh.

Revascularization procedures were the most frequent post-discharge MACE in our study. While most studies report the frequency and predictors of mortality following index MI, especially STEMI [36, 37], focus on rehospitalization and revascularization procedures are warranted, as they consume significant healthcare resources and affect patient quality of life [38, 39]. In our study, no patient underwent primary PCI. This may be because of prolonged symptom onset to hospital arrival time [15]. Revascularization procedures are frequently delayed in Bangladesh, where the majority (67%) of healthcare expenditure is out-of-pocket [40]. Most revascularization procedures are often delayed during which patients and family secure financial resources for the intervention, a practice not compatible with treatment guidelines for acute MI of the European Society of Cardiology [41].

Studies have documented that MACE and mortality after MI is higher in females than in males worldwide, and women are less likely to receive optimal treatment, including post-discharge preventive medication even in high-income settings [2, 42, 43]. A higher rate of MACE and mortality among women has been attributed to biological sex differences and gender differences influenced by social, environmental, and community factors [44, 45]. In a large registry-based study from 125 centres in India, only 22.6% of patients with acute coronary syndrome were female [46]. There were few female patients (7%) in our study. This underrepresentation of females as participants was also a factor in previous studies conducted in Bangladesh and neighboring countries, and represents the known lower risk of cardiovascular disease in women prior to menopause [15, 47–50]. Women tend to show atypical symptoms of acute coronary events and are less likely to present with chest pain [51]. Hence, they are less likely to seek hospital care [52] and optimal treatment [53]. According to the Bangladesh demographic and health survey report of 2014, only 14.1% of women have decision-making capacity with respect to their own healthcare, and three in ten women reported that their husband is the main decision maker for their healthcare [54]. Women are underrepresented in cardiology studies and our findings are a stark reminder that female sex should be considered in designing and analyzing future studies [55].

Social and cultural factors may also explain delayed hospitalization or not seeking healthcare. A quarter of our study participants had no formal education, and 41% had education only at the primary level (Table 1). Prevous studies in Bangladesh have documented a low level of education limiting access to healthcare and negatively affecting health care seeking decisions [56, 57].

We found an elevated risk of MACE among patients aged ≥ 65 years that did not reach statistical significance, most likely because of the low numbers of such patients (13%) in our cohort. Nevertheless, increasing age is considered a significant risk factor for mortality after an acute myocardial infarction [58–60]. The choice of treatment for the elderly should be determined by early clinical assessment, time of presentation after STEMI, and underlying comorbidities [61].

We also found 25% of our respondents with DM to have a numerically higher risk of MACE, but it did not reach statistical significance. The role of DM in ACS deserves much clinical and therapeutic attention. The inflammatory status and altered glucose homeostasis with DM could cause endothelial dysfunction [62, 63] even in the absence of significant coronary artery stenoses [63] such as in the condition of acute myocardial infarction specifically for patients with multi-vessel coronary stenosis [64] and high thrombus burden in STEMI [65]. Furthermore, altered endothelial function may result in restenosis after revascularization by PCI [66] and may sustain a high thrombus burden [67]. Control of inflammatory status could be an appropriate therapeutic option to reduce the burden of cardiovascular disease [62]. Hypoglycemic drugs with anti-inflammatory properties may ameliorate conditions by directly stabilizing coronary plaques [64]. Even after STEMI and guideline-recommended treatment, glycemic control not only has shown to reduce thrombus burden [68] but also improve myocardial repair [69].

We found a statistically significant higher risk of 30-day MACE among patients who had a history of heart failure before the recent STEMI. Heart failure, together with MI, has been considered a major driver of morbidity and mortality. With an established contribution of heart failure to morbidity and mortality after MI, early risk stratification through clinical and laboratory assessment, together with preventative therapeutic strategies, is required to reduce in-hospital and 30-day MACE [70].

Several limitations warrant attention while interpreting our study findings. First, this was a relatively small observational study in a single specialized cardiac hospital in the capital city of Bangladesh, and study results may not generalize to the entire country. Further studies from multiple centers should be undertaken to ascertain rates of MACE after STEMI in larger cohorts. Secondly, in a number of patients with dyspnea a diagnosis of heart failure was likely missed due to strained resources and this might explain the low number of patients diagnosed with heart failure available for our analyses. Thirdly, data of the 30-day follow-up was obtained through phone calls by study physicians with no documented evidence of patients’ adherence to post-discharge therapy and of MACE outcomes to verify responses from patients and or family members. In addition, troponin levels were not available for most partipants. In future studies, review of records of the treatment provided is recommended in determining MACE outcomes. Despite these limitations, this research offers essential information of in-hospital and 30-day MACE after STEMI in Bangladesh.

## Conclusions

The study shows a considerable risk of in-hospital and 30-day MACE occurring in patients referred with STEMI in Bangladesh. Our findings highlight the need for resources to provide guideline-recommended treatment for patients with myocardial infarction.

## Data Availability

Data generated during the study are subject to a data access policy of icddr,b and are available from icddrb’s research administration on reasonable request through the corresponding author.

## Abbreviations

ASA: Acetylsalicylic acid
ACE inhibitor: Angiotensin converting enzyme inhibitor
BMI: Body mass index
BP: Blood pressure
CABG: Cronary artery bypass grafting
CI: Confidence intervals
cTn-I: Cardiac troponin I
DM: Diabetes mellitus
ECG: Electrocardiogram
HbA1c: Glycated hemoglobin
HDL: High-density lipoprotein
HR: Hazard ratio
LDL: Low-density lipoprotein
MACE: Major adverse cardiac events
MI: Myocardial infarction
NICVD: National Institute of Cardiovascular Disease
PCI: Percutaneous coronary intervention
STEMI: ST-segment elevation myocardial infarction
